# Identifying misdiagnosed bipolar disorder using support vector machine: feature selection based on fMRI of follow-up confirmed affective disorders

**DOI:** 10.1038/s41398-023-02703-z

**Published:** 2024-01-08

**Authors:** Xiaowei Jiang, Bo Cao, Chao Li, Linna Jia, Yi Jing, Wei Cai, Wenhui Zhao, Qikun Sun, Feng Wu, Lingtao Kong, Yanqing Tang

**Affiliations:** 1 Brain Function Research Section, Department of Radiology, The First Hospital of China Medical University, Shenyang, Liaoning 110001 PR China; 2 Department of Psychiatry, Faculty of Medicine & Dentistry, University of Alberta, Edmonton, AB T6G 2B7 Canada; 3 Department of Psychiatry, The First Hospital of China Medical University, Shenyang, Liaoning 110001 PR China; 4 Neusoft Research of Intelligent Healthcare Technology, Co. Ltd, Shenyang, Liaoning 110167 PR China; 5 Department of Radiation Oncology, The First Hospital of China Medical University, Shenyang, Liaoning 110001 PR China; 6 Department of Geriatric Medicine, The First Hospital of China Medical University, Shenyang, Liaoning 110001 PR China

**Keywords:** Bipolar disorder, Diagnostic markers, Predictive markers

## Abstract

Nearly a quarter of bipolar disorder (BD) patients were misdiagnosed as major depressive disorder (MDD) patients, which cannot be corrected until mania/hypomania develops. It is important to recognize these obstacles so that the appropriate treatment can be initiated. Thus, we sought to distinguish patients with BD from MDD, especially to identify misdiagnosed BD before mania/hypomania, and further explore potential trait features that allow accurate differential diagnosis independent of state matters. Functional magnetic resonance imaging scans were performed at baseline on 92 MDD patients and 48 BD patients. The MDD patients were then followed up for more than two years. After follow-up, 23 patients transformed into BD (tBD), and 69 patients whose diagnoses remained unchanged were eligible for unipolar depression (UD). A support vector machine classifier was trained on the amygdala-based functional connectivity (FC) of 48 BD and 50 UD patients using a novel region-based feature selection. Then, the classifier was tested on the dataset, encompassing tBD and the remaining UD. It performed well for known BD and UD and can also distinguish tBD from UD with an accuracy of 81%, sensitivity of 82.6%, specificity of 79%, and AUC of 74.6%, respectively. Feature selection results revealed that ten regions within the cortico-limbic neural circuit contributed most to classification. Furthermore, in the FC comparisons among diseases, BD and tBD shared almost overlapped FC patterns in the cortico-limbic neural circuit, and both of them presented pronounced differences in most regions within the circuit compared with UD. The FC values of the most discriminating brain regions had no prominent correlations with the severity of depression, anxiety, and mania/hypomania (FDR correction). It suggests that BD possesses some trait features in the cortico-limbic neural circuit, rendering it dichotomized by the classifier based on known-diagnosis data.

## Introduction

Bipolar disorder (BD) is one of the most serious affective disorders, which affects about 45 million people in the world [1] and shares overlapping depressive symptoms with major depressive disorder (MDD). Typically, a depressive episode precedes the onset of manic/hypomanic episode and dominates the clinical course of BD patients [2]. These clinical characteristics add ambiguity to the categorization of BD and MDD, and thus, misdiagnosis, inappropriate treatment, and poor prognosis frequently occur [3, 4]. However, BD and MDD are categorized by the characteristics of symptomatology rather than the biomarkers, and the discriminative physiopathology mechanism before manic/hypomanic episodes is unclear.

Resting-state functional magnetic resonance imaging (rs-fMRI) technology enables objective neuroimaging markers for the study of affective disorders by observing the functions of live brains. Prior studies have shown that rs-fMRI can distinguish the two affective disorders [5, 6] as well as sensitively detect variations between subthreshold BD and MDD [7]. Furthermore, by combining rs-fMRI with follow-up observation, several studies even distinguished patients who were initially diagnosed with MDD and transformed into BD after manic/hypomanic appearance in the subsequent follow-up (tBD) from MDD patients. In these studies, tBD patients showed specific functional connectivity (FC), spontaneous neural activity, and gray matter volume in cortical regions, including the frontal, temporal, occipital, parietal, insular regions, and limbic system, including the amygdala [8–10]. It has been postulated that cortico-limbic connectivity is associated with neuroimaging mechanisms related to BD [11, 12] and MDD [13, 14]. Amygdala, as one of the major nodes of the cortico-limbic neural circuit, connects to various regions within the neural circuit [15]. The disruptive amygdala-based FC within the cortico-limbic neural circuit may serve as a candidate neuroimaging marker for distinguishing between BD and MDD [16, 17], as well as developing the risk of BD [18]. Nevertheless, the results of such studies at the group level cannot be implemented for differential diagnosis at the individual level. The clinical translation of the research findings is urgently required in order to help clinicians diagnose and treat BD patients correctly and promptly.

The extensive application of machine learning in the medical field has lately made it possible to translate scientific results into clinical practice. Researchers established a model that can distinguish various diseases at the individual level and applied the model to the new data for classification or prediction. Support vector machine (SVM), a supervised machine learning method, is designed to identify the spatially distributed model of brain alterations across multiple voxels [19]. It has been used to examine the brain alterations between patients with MDD and BD on rs-fMRI data with accuracy ranging from 86% to 90.89%. Furthermore, the regions involving the insular, cerebellar, pallidum, angular, parietal, frontal, occipital, and temporal cortices contributed to the classification [20, 21]. Regarding the early identification rs-MRI studies of MDD and BD, one study involving 33 tBD patients, 33 unipolar disorder (UD) patients who remained depressed for over 5 years, 33 BD patients, and 33 controls trained a classifier on tBD and UD patients with an accuracy of 78.13%. The study further explored the functional abnormalities among the three patient groups, and the differences were primarily in the somatomotor network, default-mode network, and cognitive control network [22]. A recent study utilizing clinical features and functional activities of the reward circuit differentiated patients with tBD from those with UD with an accuracy of 87.5% [23]. On the clinical side, two recent studies adopted clinical variables such as demographic information and medical records, as well as oxidative stress biomarkers, to build prediction models of BD [24, 25]. As mentioned, the supervised machine learning methods allow early identification of the two affective disorders and even the prediction of bipolar conversion.

Mary L. Phillips and Eduard Vieta have pointed out the salient meaning of the trait markers, which refer to functional disturbances in the neural circuit that may persist through all the states of BD, in understanding the pathophysiologic mechanisms of BD [26]. Indeed, numerous functional neuroimaging studies have supported trait-related deficits in BD across depressive, manic, hypomanic, and euthymic states [27–31]. These trait features appear to aid in the early differentiation between MDD and BD.

Accordingly, we aim to establish a SVM classifier to distinguish patients with BD from MDD, especially those misdiagnosed with BD before manic/hypomanic episode. Further, we intend to find potential trait features by using a novel region-based feature selection that allows accurate differential diagnosis regardless of the states of the affective disorders.

## Methods and materials

### Subjects

**Fig. 1:**
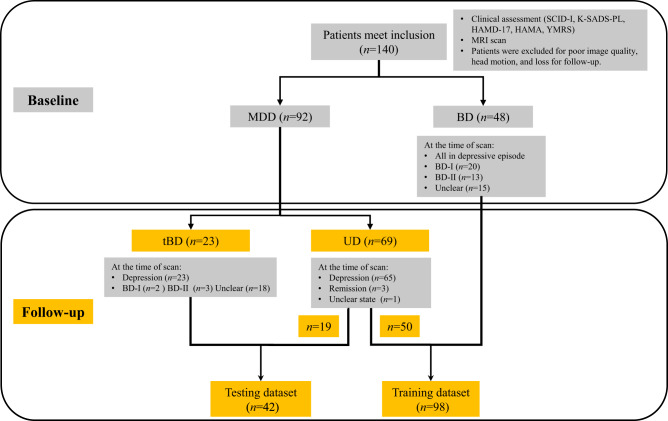
The grouping flow chart. UD unipolar depression (patients with major depressive disorder whose diagnosis remained unchanged after follow-up), BD bipolar disorder, tBD patients who initially diagnosed with major depressive disorder transformed into BD during follow‐up, SCID-I Structured Clinical Interview for DSM-IV Axis I Disorders, K-SADS-PL Schedule for Affective Disorders and Schizophrenia for School-age Children-present and Lifetime Version, HAMD-17 17-item Hamilton Depression Rating Scale, HAMA Hamilton Anxiety Scale, YMRS Young Mania Rating Scale.

Finally, a total of 140 patients from the Department of Psychiatry of the First Hospital of China Medical University were included in this study. Figure 1 illustrates the patient grouping process from enrollment to regrouping after follow-up.

At baseline, 92 patients were diagnosed with MDD and 48 with BD. The patients were recruited and underwent MRI scans from February 2009 to July 2019. MDD patients were then followed up every six months on average from October 2009 to November 2020. The manic/hypomanic symptom items in the Structured Clinical Interview for DSM-IV, Axis I Disorders (SCID-I) [32] served as the basis for the follow-up process. The process of assessment was completed by at least one researcher who had passed a consistency test. If a patient exhibited a suspicious manic/hypomanic episode during the follow-up, we would recommend the patient visit the outpatient clinic once more. A chief physician would confirm the diagnosis in a face-to-face interview on the condition that the patients agreed to return. Otherwise, a chief physician would complete the confirmation of the diagnosis on a call. By the time of November 2020, 23 patients who were diagnosed with MDD at baseline were eventually diagnosed with tBD. The diagnosis of the other 69 MDD patients remained unchanged, and they were assigned to the UD group.

All patients, ranging from 13 to 55 years old, met either MDD or BD diagnostic criteria by using SCID-I for adult patients or Schedule for Affective Disorders and Schizophrenia for School-age Children-Present and Lifetime Version (K-SADS-PL) for minor patients [33]. Additionally, the 17-item Hamilton Depression Rating Scale (HAMD-17) [34], the Hamilton Anxiety Scale (HAMA) [35], and the Young Mania Rating Scale (YMRS) [36] were employed to assess the clinical symptom severity. Professional psychiatrists carried out clinical evaluations at the psychiatric outpatient clinic of the First Hospital of China Medical University, and well-trained researchers who had passed the clinical evaluation consistency test further validated them at the Brain Function Research Section.

Patients were excluded if they had: (1) any forms of nonpharmacological treatments before the scan, such as electroconvulsive therapy (ECT) and transcranial magnetic stimulation (TMS); (2) current or past history of other DSM-IV Axis I or II disorders; (3) neurological diseases involving epilepsy, neurodegenerative disease, cerebrovascular disease, brain tumors, and head trauma with a loss of consciousness over five minutes; (4) serious somatic diseases, particularly diseases that may cause changes in brain tissue, such as uncontrolled diabetes and hypertension; (5) claustrophobia; (6) MRI contraindications; and (7) pregnancy.

Prior to study enrollment, all participants willingly submitted their written informed consent. If they were under 18, their guardians additionally provided informed consent on their behalf. This study was approved by the Medical Science Research Ethics Committee of the First Hospital of China Medical University.

### MRI acquisition

All the MRI scans were conducted at baseline using a GE Signa HDX 3.0 T scanner with an 8-channel head coil at the Department of Radiology of the First Hospital of China Medical University. During the scan, patients were instructed to close their eyes, stay awake, and try not to think. Earplugs and foam pads were utilized to reduce noise and head motion. The echo-planar imaging sequence was performed to get the resting-state functional MRI (rs-fMRI) data with the following parameters: repetition time (TR) = 2000 ms, echo time (TE) = 40 ms, field of view (FOV) = 240 mm × 240 mm, image matrix size = 64 × 64, voxel size = 3.75 × 3.75 × 3 mm^3^, flip angle = 90°, 35 slices with slice thickness = 3 mm, spacing between slices = 3 mm, and scan time = 400 s.

### MRI preprocessing

Functional image preprocessing was carried out utilizing techniques implemented in the SPM8-based Data Processing Assistant for Resting-State fMRI Advanced Edition (DPARSFA) toolbox on the MATLAB 2010b platform (PC version) [37]. The first ten time points were removed to minimize equilibration effects that could distort the data. The remaining images underwent slice-timing correction and motion realignment. Patients whose head motion exceeded 3 mm of translation or 3 degrees of rotation were excluded. There were no statistical differences in FD values among UD, tBD, and BD. Then spatial normalization to standard Montreal Neurological Institute (MNI) space was applied with a resolution of 3 mm × 3 mm × 3 mm, followed by spatial smoothing (6 mm × 6 mm × 6 mm full width at half maximum Gaussian Kernal). Further preprocessing steps included linear detrending, nuisance regression with white matter signal, cerebrospinal fluid signal, global signal, and head motion (Rigid-body 6), as well as temporal bandpass filtering (0.01–0.08 Hz) before subjecting the images to FC analysis.

### FC analysis

Analysis of FC was also performed using the DPABI toolbox. The bilateral amygdalae, based on the automatic anatomical labeling (AAL) atlas [38], were chosen as the seeds of FC analysis. The Pearson correlations between the mean time series of the bilateral amygdala and the remaining whole-brain voxels (all in gray matter) were calculated. Finally, the correlation coefficients were transformed into z scores by a Fisher’s z-transformation. This process yielded a 61 × 73 × 61 FC image, which is the default setting of DPABI to minimize the differences in voxel numbers before and after normalization. The image was then transformed into a vectorial feature space for each individual. Thus, we got 61 × 73 × 61 dimensional features and used them for subsequent classification analysis.

### Statistical analyses

#### Demographic and clinical characteristics

We performed statistical analyses using SPSS 24.0 software (SPSS Inc., Chicago, Illinois). For continuous variables containing age, years of education, duration of illness, symptom scales (HAMD-17, HAMA, and YMRS), and FD values, comparisons were conducted by one-way analysis of variance (ANOVA) for normally distributed variables or the Kruskal-Wallis H test for non-normally distributed variables. Categorical variables comprising sex, first-episode, and medication were analyzed by the Chi-square test or Fisher’s exact probability test. The continuous variables were expressed as median and interquartile range (IQR) or mean ± standard deviation according to the data distribution. Statistical significance was set at a *p* value <0.05 in all comparisons.

#### SVM analysis

The SVM algorithm was applied as the classifier due to its excellent performance in a small sample size [39] by adopting the Python-based Scikit-learn package (http://scikit-learn.org). The primary steps consisted of: (i) initial selecting features; (ii) further selecting discriminative brain regions; (iii) training a classifier; and (iv) evaluating the classifier.

(i) Initial selecting features: Random Forest (RF) is an embedded-type feature selection method that has been shown to significantly improve model performance [40–42], is commonly used in data mining [43], and recognizes important genes [44]. Mean decrease impurity (MDI) is a method to measure the feature importance in RF model. It is the average value of the decrease in node impurity (weighted by the probability of reaching that node) over all trees. In this study, we applied a supervised learning approach and built an RF model with default parameters on the training set. The importance score for each voxel was obtained by MDI, which can retain non-zero features.

Additionally, we used a SHAP analysis to recompute the importance score of each voxel and validate the reliability of the feature selection by MDI (Supplementary Table 1).

(ii) Further selecting discriminative brain regions: This process is based on the preliminary features acquired in the training set, as described above. For each brain region defined by the AAL atlas, the feature importance score is the total score of the voxel located in the corresponding region. Thus, the brain regions can be ranked in descending order according to their contributions, generating a sorted list of brain regions. Then, based on the list, we calculated the correlation between the mean FC and the group label for each brain region using the *U* test and selected brain regions until the *p* value was higher than 0.05. Finally, the features whose importance score was not equal to 0 in discriminative brain regions were chosen.

Regarding feature selection, we also tried other methods, such as univariate analysis (Variance), multivariate analysis (Pearson, ANOVA, Chi-square), and the L1 regularization penalty term. We present the procedure and results in Supplementary Tables 2 and 3.

(iii) Training a classifier: The SVM classifier was trained to distinguish between UD and BD/tBD. We selected the linear function as the kernel function with the default settings, and then the coefficient of the penalty term was adjusted by the grid search method. We also selected the features from the training set that showed differences between the BD and MDD groups as classification features using the feature selection method described above.

Additionally, logistic regression classifiers were also adopted as comparison methods, and details of analyses and results are provided in Supplementary Tables 4 and 5.

(iv) Evaluating the classifier: To validate and evaluate the performance of the classifier, eightfold cross-validation was used on the training sample (50 UD patients and 48 BD patients). In k-fold cross-validation, when the K value is small, the model is prone to overfitting with low deviation and high variance; as the K value increases, the deviation increases and the variance decreases. Therefore, we employed cross-validation with *K* = 8 to balance deviation and variance in this study. The average accuracy, sensitivity, specificity, receiver operating characteristics curve (ROC), and area under the ROC curve (AUC) of these eight tests were reported as the main evaluation indicators of the models.

As for the generalization and prediction performance evaluation, the established classifier was adopted on an independent testing sample of 19 UD patients and 23 tBD patients. Accuracy, sensitivity, specificity, ROC, and AUC were also obtained during this test. To test the generalization performance of the classifier on external data, we also adopted two public datasets, including MDD and BD patients. The details are shown in Supplementary Table 6 and Supplementary Fig. 1.

The permutation tests were utilized to evaluate the statistical significance of classification accuracy. We randomly selected data from training and testing datasets 1000 times and calculated the classification accuracy of each iteration with eightfold cross-validation. A *p* value less than 0.05 was defined as significant.

#### Comparisons of FC across the three groups

To further investigate differences in FC among the three groups in the discriminative brain regions, we listed 10 brain regions with the highest feature importance scores and extracted the mean FC value for each of them. ANOVA was conducted to compare the FC value in each brain region among UD, tBD, and BD. The post-hoc analyses were performed by Bonferroni or Tamhane tests according to the homogeneity of variance. A *p* value < 0.05 was considered statistically significant.

#### Correlation analyses between FC and symptom scales

In order to investigate the potential clinical values of these regions, Pearson’s correlation analysis was applied to determine the association between the mean FC value of each brain region and each symptom score (HAMD-17, HAMA, and YMRS), respectively, including the ten most contribution brain regions. Results were corrected using the Benjamini–Hochberg false discovery rate (FDR) (*q* < 0.05) method.

## Results

### Demographic and clinical characteristics

No significant differences were found in age, sex, education year, and FD among the UD, tBD, and BD groups (*p* > 0.05). However, illness duration, first episode, medication, and symptom scales (HAMD-17, HAMA, and YMRS) were statistically different among these three groups (*p* < 0.05). The median follow-up time was 54.5 months (IQR: [23.75 months; 79.25 months]) for the UD group and 23 months (IQR: [2 months; 44 months]) for the tBD group. Table 1 contains more detailed information on demographic and clinical characteristics. Supplementary Table 7 contains the characteristics of the training and testing datasets.

**Table 1:** Clinical characteristics of UD, tBD, and BD.

Variables	UD (*n* = 69)	tBD (*n* = 23)	BD (*n* = 48)	*F*/χ^2^/H	*p*
Age, year (Md, IQR)	29.0, 17.5	27.0, 13.0	24.5, 11.5	2.419^a^	0.298^a^
Sex (female/male)	43/26	14/9	30/18	0.019^c^	0.990^c^
Education year (Md, IQR)	12.0, 6.0	12.0, 6.0	13.0, 5.0	3.245^a^	0.197^a^
Illness duration, month (Md, IQR)	9.0, 22.8	9.2, 19.9	33.8, 52.5	19.151^a^	0.000^a^ *
First-episode (Y/N/unclear)	59/3/7	20/2/1	18/28/2	47.940^d^	0.000^d^ *
Medication (Y/N)	24/45	8/15	34/14	16.452^c^	0.000^c^ *
HAMD-17 score (M ± SD)	24.6 ± 8.1	24.3 ± 6.7	15.6 ± 7.3	21.627^b^	0.000^b^ *
HAMA score (Md, IQR)	17.0, 11.0	24.0, 13.0	11.5, 9.5	16.841^a^	0.000^a^ *
YMRS score (Md, IQR)	0.0, 2.0	0.0, 0.0	2.0, 5.0	9.044^a^	0.011^a^ *
Follow-up time, month (Md, IQR)	54.5, 55.5	23.0, 42.0	NA	NA	NA
FD (Md, IQR)	0.089, 0.075	0.097, 0.049	0.100, 0.050	1.503^a^	0.472^a^

*Md* median, *IQR* interquartile range, *M* mean, *SD* standard deviation, *HAMD-17* 17-item Hamilton Depression Rating Scale, *HAMA* Hamilton Anxiety Scale, *YMRS* Young Mania Rating Scale, *FD* framewise displacement, *UD* unipolar depression (patients with major depressive disorder whose diagnosis remains unchanged after follow-up), *BD* bipolar disorder, *tBD* patients who initially diagnosed with major depressive disorder transformed into BD during follow‐up; *p < 0.05 was considered a statistical difference, *NA* not available.

^a^Kruskal-Wallis *H* test.

^b^ANOVA test.

^c^Chi-square test.

^d^Fisher’s exact test.

### SVM classifier results

**Fig. 2:**
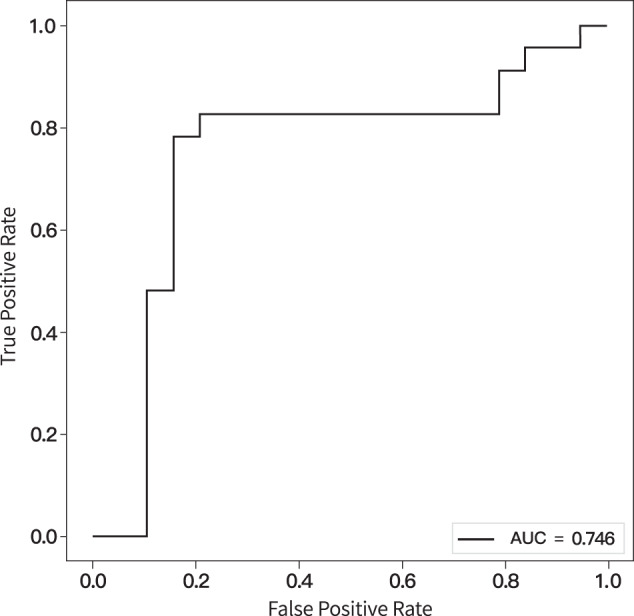
The ROC curve of SVM classifier performance in the testing dataset. ROC receiver operating characteristics, AUC area under the curve.

In the classification of UD and tBD groups, namely in the testing dataset, the accuracy, sensitivity, and specificity were 81%, 82.6%, and 79%, respectively (permutation test, *p* = 0.004). The ROC curve with an AUC value of 74.6% is displayed in Fig. 2. In the classification of the training dataset, the mean accuracy, sensitivity, and specificity were 73.5%, 75%, and 72%, respectively (permutation test, *p* = 0.029). A mean ROC curve with an AUC value of 73.5% was also obtained (Supplementary Fig. 2).

### Brain regions contributed most to classification

**Fig. 3:**
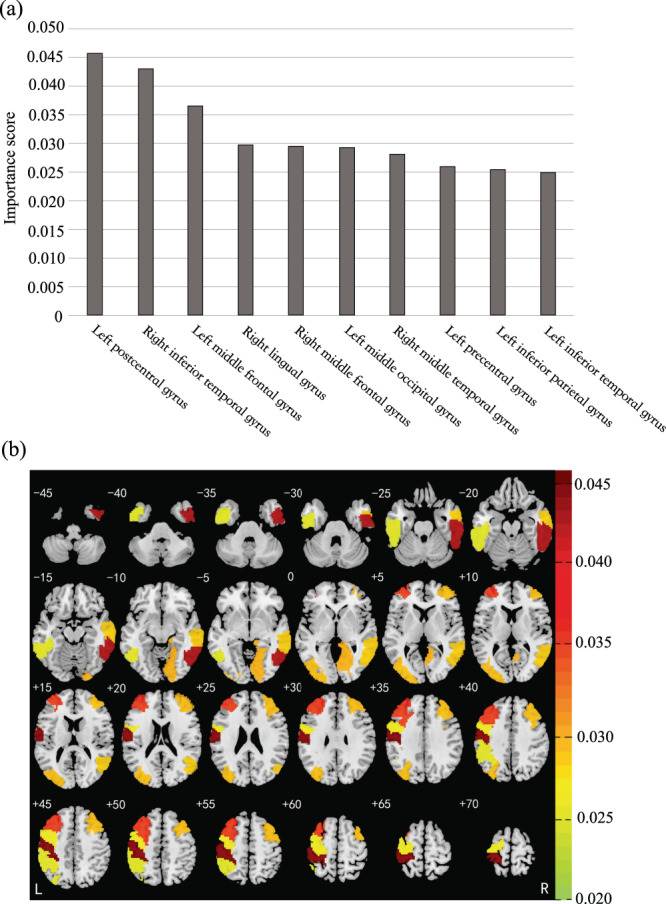
Brain regions contributed most to classification. **a** The importance scores of the first ten regions. The *X* axis represents the first ten regions contributing most to classification. The *Y* axis represents the feature importance score per brain region. **b** Regions within the cortico-limbic neural circuit contributed most to classification. The color bar represents feature importance score for classification. The number corresponds to z-coordinate; L left, R right.

The feature importance scores of the first ten brain regions contributing most to classification is displayed in Fig. 3a. The regions in order of importance were the left postcentral gyrus, the right inferior temporal gyrus, the left middle frontal gyrus, the right lingual gyrus, the right middle frontal gyrus, the left middle occipital gyrus, the right middle temporal gyrus, the left precentral gyrus, the left inferior parietal gyrus, and the left inferior temporal gyrus (Fig. 3b).

### Differences in FC across the three groups

**Fig. 4:**
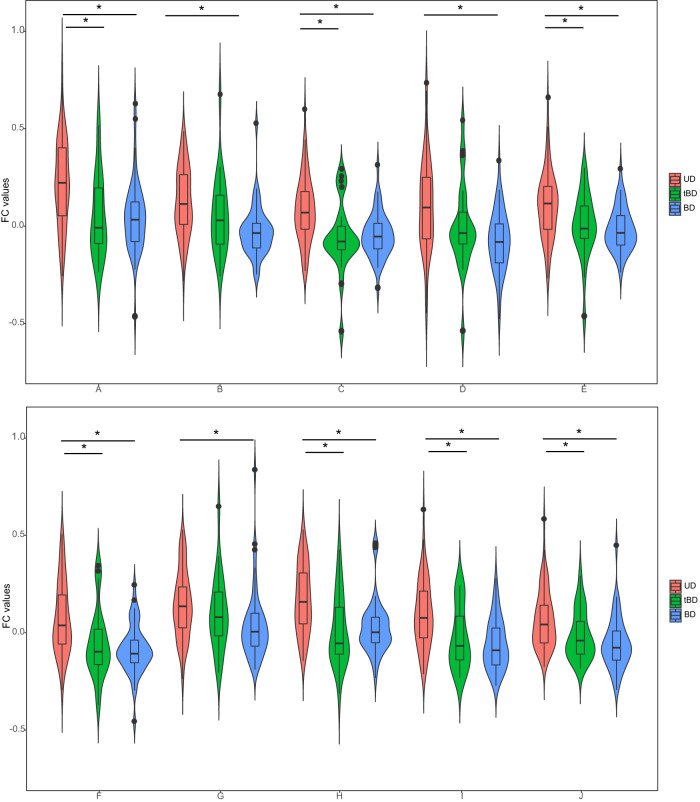
FC differences among UD, tBD, and BD groups in the ten brain regions. UD unipolar depression (patients with major depressive disorder whose diagnosis remained unchanged after follow-up), BD bipolar disorder, tBD patients who initially diagnosed with major depressive disorder transformed into BD during follow‐up; the *X* axis refers to the top ten brain regions that contributed most to classifying BD/tBD and UD: A, the left postcentral gyrus; B, the right inferior temporal gyrus; C, the left middle frontal gyrus; D, the right lingual gyrus; E, the right middle frontal gyrus; F, the left middle occipital gyrus; G, the right middle temporal gyrus; H, the left precentral gyrus; I, the left inferior parietal gyrus; J, the left inferior temporal gyrus; the *Y* axis refers to mean amygdala-based FC values extracted from the brain regions with important features; *p < 0.05 was considered a significant difference (two-tailed).

Here, we chose the first ten informative brain regions for classification to perform the FC comparisons among the UD, tBD, and BD groups. The results showed that BD and tBD did not differ in ten regions, while significant differences were observed between UD and BD (UD < BD; *p* < 0.05, two-tailed). When it comes to UD and tBD, differences were also observed in these regions (UD < tBD; *p* < 0.05, two-tailed) except in the inferior temporal gyrus, lingual gyrus, and middle temporal gyrus of the right cerebrum (*p* > 0.05, two-tailed) (Table 2, Figs. 3b and 4).

**Table 2:** FC differences in the most contribution brain regions among UD, tBD, and BD groups.

The serial number	Brain regions	Hemisphere	*F*	*p*	Post-hoc analysis
	Postcentral gyrus		11.272	<0.001	UD < BD (*p* < 0.001*)
A		Left			UD < tBD (*p* = 0.004*)
					tBD vs. BD (*p* = 1.000)
	Inferior temporal gyrus		12.437	<0.001	UD < BD (*p* < 0.001*)
B		Right			UD vs. tBD (*p* = 0.227)
					tBD vs. BD (*p* = 0.145)
	Middle frontal gyrus		13.333	<0.001	UD < BD (*p* < 0.001*)
C		Left			UD < tBD (*p* = 0.001*)
					tBD vs. BD (*p* = 1.000)
	Lingual gyrus		10.998	<0.001	UD < BD (*p* < 0.001*)
D		Right			UD vs. tBD (*p* = 0.097)
					tBD vs. BD (*p* = 0.506)
	Middle frontal gyrus		10.418	<0.001	UD < BD (*p* < 0.001*)
E		Right			UD < tBD (*p* = 0.015*)
					tBD vs. BD (*p* = 1.000)
	Middle occipital gyrus		14.673	<0.001	UD < BD (*p* < 0.001*)
F		Left			UD < tBD (*p* = 0.005*)
					tBD vs. BD (*p* = 1.000)
	Middle temporal gyrus		4.485	0.013	UD < BD (*p* = 0.010*)
G		Right			UD vs. tBD (*p* = 1.000)
					tBD vs. BD (*p* = 0.585)
	Precentral gyrus		14.663	<0.001	UD < BD (*p* < 0.001*)
H		Left			UD < tBD (*p* < 0.001*)
					tBD vs. BD (*p* = 1.000)
	Inferior parietal gyrus		15.246	<0.001	UD < BD (*p* < 0.001*)
I		Left			UD < tBD (*p* = 0.005*)
					tBD vs. BD (*p* = 1.000)
	Inferior temporal gyrus		11.390	<0.001	UD < BD (*p* < 0.001*)
J		Left			UD < tBD (*p* = 0.046*)
					tBD vs. BD (*p* = 0.782)

*UD* unipolar depression (patients with major depressive disorder whose diagnosis remains unchanged after follow-up), *BD* bipolar disorder, *tBD* patients who initially diagnosed with major depressive disorder transformed into BD during follow-up, vs. versus.

*p < 0.05 was considered a statistical difference.

### Correlations between FC and symptom scales

The Pearson correlation analysis revealed that there was a negative correlation between the HAMA total score and the left postcentral gyrus in patients with BD (*r* = −0.328, *p* = 0.023). The total score of YMRS in tBD had negative correlations with the left middle frontal gyrus (*r* = −0.551, *p* = 0.015) and the left precentral gyrus (*r* = 0.517, *p* = 0.023), but positive correlations with the right inferior temporal gyrus (*r* = 0.501, *p* = 0.029) and the right middle temporal gyrus (*r* = 0.467, *p* = 0.044). However, no significant correlations survived between FC and symptom scales (HAMD-17, HAMA, and YMRS) after the FDR correction (Supplementary Table 8).

## Discussion

To the best of our knowledge, this is the first study that used amygdala-based FC to train a classifier based on an innovative region-based feature selection method to separate BD and MDD, especially to screen tBD. Besides, the FC comparisons showed that BD and tBD shared an almost overlapped FC pattern in the cortico-limbic neural circuit, and both of them presented pronounced differences in a majority of regions within the circuit relative to UD. Meanwhile, the most discriminative brain regions located in the cortico-limbic neural circuit were irrelevant to state matters, which included the severity of depression, anxiety, and manic/hypomanic symptoms in the short term. The findings indicated that the misdiagnosed BD could be dichotomized by the classifier using FC data from follow-up confirmed affective disorders. In other words, BD may possess some FC features that are not related to the state but rather to the nature of the disease. The findings may contribute to developing a tool to ameliorate the dilemma of differential diagnosis and could provide a new perspective for predicting affective disorders.

In the present study, the classifier has a high sensitivity to identify the misdiagnosed BD patients who were diagnosed with MDD by experienced psychiatrists before their first manic/hypomanic episode. Only a limited number of studies generalize a classifier to external samples or uncertain patients. A two-center cross-sectional study found that a model with good performance, which was trained by one center, was able to differentiate the two disorders from another center [45]. Another study trained a classifier with high accuracy based on the confirmed diagnoses of BD, MDD, and healthy control groups. The classifier was then applied to patients with ambiguous diagnoses and categorized the majority of patients (91.7%) by referencing medication response [46]. The evidence implies that a classifier established by patients with known-diagnosis is available for application to patients with obscure diagnoses. One of the possible reasons for the result can be explained by the trait features, which were inferred from two other findings of the study. Firstly, in the cortico-limbic neural circuit, tBD patients shared more similar FC patterns with BD, which is in agreement with the findings of Shao et al. [22]. In addition, both tBD and BD can be robustly separated from UD in most regions of the neural circuit. The results fit well with Jiang et al. [9, 10, 47] and Zhang et al. [48], who reported discrepant functions between UD and BD/tBD within the cortico-limbic neural circuit by using different rs-fMRI approaches. Secondly, the negative correlations between FC and symptom severity again imply that the FC features within the cortico-limbic neural circuit may be unaffected by states, which is in line with several previous studies. For instance, similar functional changes of the striatal areas and inferior parietal gyrus were observed in depressive and euthymic BD patients, leading authors to hypothesize that the functional alterations of cortical-striatal neural circuits were a trait-like alteration unrelated to mood states [49]. Both manic and euthymic patients with BD exhibited a declining gray matter volume trend in the left hippocampus, parahippocampal gyrus, and amygdala and raised gray matter volume in the left orbitofrontal cortex. Meanwhile, there were no prominent distinctions between the mania and euthymia groups [50]. Additionally, functional abnormalities in the regions of the cortico-limbic neural circuit were seen in both BD patients who had recently experienced a manic episode and those who had never experienced manic/hypomanic symptoms [8–10, 51]. Consequently, functional alterations within the cortico-limbic neural circuit may be the trait neuroimaging markers of patients with BD who either develop manic/hypomanic symptoms or not. Along with our findings, we suppose the trait neuroimaging indicators enable classifiers to distinguish the two affective disorders and even predict the onset of affective disorders.

Feature selection is considered essential in the context of neuroimaging data-based classification. We utilized the region-based feature selection method to capture an optimal subset of brain regions for BD/tBD-UD discrimination and improve model interpretability. Jie et al. employed a similar feature selection method and obtained a preferred classification performance [21]. In addition, the optimal features spreading over the cortico-limbic neural circuit with the amygdala as a core play a major role in emotional processing [52]. In the neural circuit, there are extensive connections between cortices and limbic regions encompassing the amygdala; cortical regions send signals to limbic regions and integrate information from the limbic system. Meanwhile, emotions are associated with cognition and behavior via the limbic system [53]. The present findings and related studies consider the cortico-limbic brain circuit as a potential neural circuit for differentiating the two affective disorders [47, 54]. Fung et al. adopted the morphometry indicators of the cortical regions comprised of the left precentral, inferior parietal, and right middle temporal gyri, as well as the bilateral amygdala, as features to classify patients with BD and UD, and the classification accuracy was 74.3% [55]. Furthermore, BD and UD could be well separated through the activities of the frontal and temporal regions, as well as the amygdala elicited by tasks [56–58]. The two disorders also showed distinctive patterns such as dynamic FC, cortical thickness, FC, long-range FC strength, and cortical surface area in the left postcentral, rostral middle frontal, middle occipital, inferior parietal, and precentral gyri, as well as the right lingual, middle frontal, and middle temporal gyri [17, 55, 59–63]. A recently published study on the classification of tBD and UD found an accuracy of 70% based on multiple function parameters, i.e., FC, nodal efficiency, and degree centrality of the reward circuit, and an even higher accuracy of 87.5% using a combination of functional and clinical features. Although the feature selection methods and model-building principles are different, the findings of this study suggest that incorporating clinical information can improve the performance of models for early recognition of affective disorders [23]. In summary, decent feature selection methods can yield satisfactory classification results and account for the neuroimaging mechanism in differential diagnosis at an early stage.

In conclusion, the amygdala-based FC features within the cortico-limbic neural circuit may serve as promising trait neuroimaging markers for classifying BD and MDD, even predicting BD. Likewise, the features afford underlying neuroimaging mechanisms for diagnostic distinctions.

There are several limitations that should be noted. First, the sample size was insufficiently large. Our team is still working on increasing the sample size and overcoming the restriction as of right now. Second, the sample included patients taking medication. Although the effect of medication on fMRI may have been limited [64], the results should be interpreted with caution. Finally, follow-up confirmed affective disorders from multiple centers are needed to verify the generalizability of the classifier for further clinical transformation.

### Supplementary information

Supplementary material

Supplementary figure and table legends

Supplementary Figure 1

Supplementary Figure 2

## Data Availability

The data that supports the findings of this study is available from the corresponding author upon reasonable request.
