# Supplementary material for: Identifying misdiagnosed bipolar disorder using support vector machine: feature selection based on fMRI of follow-up confirmed affective disorders
Source: Transl Psychiatry. 2024 Jan 8;14:9. doi: 10.1038/s41398-023-02703-z (PMC10774279; doi:10.1038/s41398-023-02703-z)
Supplement: Supplementary file 1 — Supplementary material [file 41398_2023_2703_MOESM1_ESM.docx]

Supplementary material

The results showed that the first ten important brain regions were the same as the primary results, except that there were slight differences in the order of importance of each brain region.

**Supplementary Table 1**

The importance scores of the first ten regions by SHAP analysis.

| Brain regions | Importance score |
| --- | --- |
| Left middle frontal gyrus | 0.027663496 |
| Right inferior temporal gyrus | 0.027499573 |
| Left middle occipital gyrus | 0.027124123 |
| Left postcentral gyrus | 0.02662882 |
| Left precentral gyrus | 0.01971729 |
| Right lingual gyrus | 0.018515776 |
| Right middle temporal gyrus | 0.018291638 |
| Left inferior temporal gyrus | 0.018206796 |
| Left inferior parietal gyrus | 0.018205375 |
| Right middle frontal gyrus | 0.016095651 |

Various results of other feature selection methods, including univariate analysis (Variance), multivariate analysis (Pearson, ANOVA, Chi-square), and L1 regularization penalty term, are displayed in Supplementary Tables 2 and 3.

**Supplementary Table 2**

Model performance of Variance, Pearson, ANOVA, and Chi-square feature selection methods.

| Feature selection | Dataset | AUC | Accuracy | Sensitivity | Specificity |
| --- | --- | --- | --- | --- | --- |
| Variance | Training dataset | 62.3% | 60.2% | 60.4% | 60.1% |
|  | Testing dataset | 65.0% | 69.1% | 60.9% | 79.0% |
| Pearson | Training dataset | 60.3% | 60.2% | 58.3% | 62.2% |
|  | Testing dataset | 70.3% | 73.8% | 69.6% | 79.0% |
| ANOVA | Training dataset | 60.7% | 61.0% | 64.5% | 58.3% |
|  | Testing dataset | 70.0% | 73.8% | 69.6% | 79.0% |
| Chi-square | Training dataset | 60.2% | 65.0% | 64.5% | 66.7% |
|  | Testing dataset | 70.3% | 76.2% | 73.9% | 79.0% |

The feature selection method based on the L1 regularization penalty term achieved similar results. In a linear model, the coefficients of the model are the weights of the features and can therefore be used to characterize the importance scores of the features. The setting of the penalty term parameter was the default parameter, which was equal to 1. Then, the steps included selecting discriminative brain regions, training a classifier on the training sample, and evaluating the generalization and prediction performance of the classifier using an independent testing sample. The results are as follows.

**Supplementary Table 3**

Model performance of L1 regularization penalty term feature selection.

| Dataset | AUC | Accuracy | Sensitivity | Specificity |
| --- | --- | --- | --- | --- |
| Training dataset | 73.5% | 72.6% | 72.9% | 72.3% |
| Testing dataset | 72.3% | 76.2% | 78.3% | 73.4% |

Least absolute shrinkage and selection operator (Lasso) and elastic net logistic regression classifiers ([https://scikit-learn.org/stable/modules/linear_model.html#](https://scikit-learn.org/stable/modules/linear_model.html)) were chosen to be compared with SVM classifier results and comparative experiments were conducted with constant feature selection.

Lasso is a linear regression method that uses L1 regularization. When dealing with categorical variables in Lasso regressions, it is common to use a grouped Lasso, which keeps together the dummy variables that correspond to a particular categorical variable as a group (i.e., if one dummy variable is selected or excluded, all the other variables in the same group are also selected or excluded). For the selection of all parameters, we determined them using the grid search method.

Elastic Net is a linear model that uses L1 and L2 as regularization matrices, and this method yields a model that is as sparse as pure Lasso regression but at the same time has the same regularization power as that provided by ridge regression. The hyperparameter of L1_ratio (regulating the specific gravity between L1 and L2) was set to 0.5 in this study.

Consistent with the method in the main text, we used 8-fold cross-validation and analyzed model performance based on AUC, accuracy, sensitivity, and specificity.

| Dataset | AUC | Accuracy | Sensitivity | Specificity |
| --- | --- | --- | --- | --- |
| Training dataset | 71.3% | 75.6% | 77.1% | 73.8% |
| Testing dataset | 73.5% | 73.8% | 82.6% | 63.1% |

**Supplementary Table 4**

Model performance of Lasso classifier.

*Note.* Lasso refers to Least Absolute Shrinkage and Selection Operator; AUC refers to area under the curve.

**Supplementary Table 5**

Model performance of elastic net classifier.

| Dataset | AUC | Accuracy | Sensitivity | Specificity |
| --- | --- | --- | --- | --- |
| Training dataset | 71.3% | 74.5% | 77.1% | 72.0% |
| Testing dataset | 72.3% | 71.4% | 73.9% | 68.4% |

*Note.* AUC refers to area under the curve.

Public rs-fMRI data for 57 patients with MDD was sourced from the SRPBS Multi-disorder MRI Dataset (unrestricted). Data used in the preparation of this work were obtained from the DecNef Project Brain Data Repository (https://bicr-resource.atr.jp/srpbsopen/), collected as part of the Japanese Strategic Research Program for the Promotion of Brain Science (SRPBS) supported by the Japanese Advanced Research and Development Programs for Medical Innovation (AMED). The dataset is available at: https://doi.org/10.7303/syn22317081 (1).

Public rs-fMRI data for 47 patients with BD was sourced from the OpenfMRI database, with the accession number ds000030. The dataset is available at: https://openfmri.org/dataset/ds000030/.

The processing of FC and SVM was conducted in accordance with the sections on FC analysis and SVM analysis in the main file.

**Supplementary Table 6**

The generalization performance of the classifier on external data.

| AUC | Accuracy | Sensitivity | Specificity |
| --- | --- | --- | --- |
| 80.7% | 74.0% | 78.7% | 70.2% |

*Note.* AUC refers to area under the curve.

**References**

1. Tanaka, S. C. et al. SRPBS Multi-disorder MRI Dataset (unrestricted). Synapse https://doi.org/10.7303/syn22317081 (2020).

**Acknowledgements**

We would like to thank the public data providers, i.e., the Japanese Strategic Research Program for the Promotion of Brain Science (SRPBS) and the OpenfMRI project.

**Supplementary Table 7**

Demographic and clinical characteristics of training and testing datasets.

| Variables | UD (*n* = 69) | | BD (*n* = 71) | | *F*/χ^2^/H | *p* |
| --- | --- | --- | --- | --- | --- | --- |
|  | UD_training (*n* = 50) | UD_testing  (*n* = 19) | BD_training  (*n* = 48) | tBD_testing  (*n* = 23) |  |  |
| Age, year  (Md, IQR) | 30.0, 18.5 | 27.0, 21.0 | 24.5, 11.5 | 27.0, 13.0 | 4.493**^a^** | 0.213**^a^** |
| Gender (male/female) | 20/30 | 6/13 | 18/30 | 9/14 | 0.434**^c^** | 0.933**^c^** |
| Education year (Md, IQR) | 12.0, 5.5 | 9.0, 6.0 | 13.0, 5.0 | 12.0, 6.0 | 5.751**^a^** | 0.124**^a^** |
| Illness duration, month (Md, IQR) | 9.0, 31.0 | 7.5, 20.3 | 33.8, 52.5 | 9.2, 19.9 | 19.943**^a^** | 0.000**^a^** * |
| First-episode (Y/N/unclear) | 43/0/7 | 16/3/0 | 18/28/2 | 20/2/1 | 55.559**^d^** | 0.000**^d^** * |
| Medication (Y/N) | 12/38 | 12/7 | 34/14 | 8/15 | 24.924**^c^** | 0.000**^c^** * |
| HAMD-17 score (M±SD) | 26.6±6.8 | 19.1±8.9 | 15.6±7.3 | 24.3±6.7 | 20.819**^b^** | 0.000**^b^** * |
| HAMA score  (Md, IQR) | 17.0, 11.0 | 14.5, 20.0 | 11.5, 9.5 | 24.0, 13.0 | 17.764**^a^** | 0.000**^a^** * |
| YMRS score  (Md, IQR) | 0.0, 1.8 | 0.0, 3.5 | 2.0, 5.0 | 0.0, 0.0 | 9.567**^a^** | 0.023**^a^** * |
| Follow-up time, month (Md, IQR) | 52.0, 71.0 | 57.5, 51.3 | NA | 23.0, 42.0 | 8.658**^a^** | 0.013**^a^** * |
| FD (Md, IQR) | 0.092, 0.072 | 0.081, 0.136 | 0.100, 0.050 | 0.097, 0.049 | 2.282**^a^** | 0.516**^a^** |

*Note.* UD, unipolar depression (patients with major depressive disorder whose diagnosis remained unchanged after follow-up); UD_training, UD patients assigned to the training dataset; UD_testing, UD patients assigned to in the testing dataset; BD, bipolar disorder; BD_training, BD patients enrolled in the training dataset; tBD_testing, patients who initially diagnosed major depressive disorder transformed into BD during follow‐up and included in the testing dataset; HAMD-17, 17-item Hamilton Depression Rating Scale; HAMA, Hamilton Anxiety Scale; YMRS, Young Mania Rating Scale; FD, framewise displacement; **p* < 0.05 was considered a statistical difference; NA, not available.

a. Kruskal-Wallis H test

b. ANOVA test

c. Chi-square test

d. Fisher’s exact test

**Supplementary Table 8**

Correlations between functional connectivity and symptom scales in the most contribution brain regions.

| Groups | Symptom scales |  | A | B | C | D | E | F | G | H | I | J |
| --- | --- | --- | --- | --- | --- | --- | --- | --- | --- | --- | --- | --- |
| UD | HAMD-17 | *r* | 0.025 | -0.048 | 0.055 | 0.034 | -0.028 | 0.1 | -0.03 | 0.001 | -0.02 | -0.093 |
|  |  | *p* | 0.841 | 0.695 | 0.655 | 0.779 | 0.819 | 0.413 | 0.807 | 0.992 | 0.873 | 0.446 |
|  | HAMA | *r* | -0.036 | -0.12 | -0.014 | 0.106 | -0.079 | 0.049 | -0.086 | -0.014 | -0.056 | -0.068 |
|  |  | *p* | 0.786 | 0.365 | 0.915 | 0.426 | 0.554 | 0.715 | 0.516 | 0.918 | 0.673 | 0.611 |
|  | YMRS | *r* | -0.113 | -0.18 | -0.115 | -0.102 | -0.256 | -0.062 | -0.16 | -0.182 | -0.097 | -0.094 |
|  |  | *p* | 0.425 | 0.203 | 0.419 | 0.472 | 0.067 | 0.661 | 0.258 | 0.197 | 0.492 | 0.509 |
| BD | HAMD-17 | *r* | -0.221 | 0.112 | 0.222 | 0.093 | 0.164 | 0.097 | 0.093 | -0.048 | -0.154 | 0.02 |
|  |  | *p* | 0.131 | 0.447 | 0.13 | 0.527 | 0.266 | 0.511 | 0.529 | 0.745 | 0.296 | 0.891 |
|  | HAMA | *r* | -0.328 | -0.043 | 0.103 | -0.212 | 0.036 | -0.089 | -0.082 | -0.215 | -0.281 | -0.023 |
|  |  | *p* | 0.023 | 0.774 | 0.486 | 0.148 | 0.808 | 0.548 | 0.58 | 0.142 | 0.053 | 0.876 |
|  | YMRS | *r* | -0.194 | 0.024 | 0.07 | -0.044 | 0.134 | 0.015 | 0.139 | -0.02 | -0.162 | -0.01 |
|  |  | *p* | 0.187 | 0.87 | 0.636 | 0.765 | 0.364 | 0.919 | 0.347 | 0.891 | 0.272 | 0.944 |
| tBD | HAMD-17 | *r* | -0.166 | -0.239 | -0.229 | -0.211 | 0.001 | -0.025 | -0.095 | -0.122 | -0.137 | -0.246 |
|  |  | *p* | 0.461 | 0.284 | 0.305 | 0.345 | 0.997 | 0.912 | 0.673 | 0.588 | 0.543 | 0.27 |
|  | HAMA | *r* | -0.211 | -0.332 | -0.131 | -0.285 | 0.04 | -0.151 | -0.386 | -0.138 | -0.252 | -0.299 |
|  |  | *p* | 0.386 | 0.165 | 0.592 | 0.237 | 0.87 | 0.538 | 0.103 | 0.572 | 0.298 | 0.214 |
|  | YMRS | *r* | -0.362 | 0.501 | -0.551 | -0.362 | -0.422 | -0.407 | 0.467 | -0.517 | -0.339 | -0.298 |
|  |  | *p* | 0.128 | 0.029 | 0.015 | 0.128 | 0.072 | 0.084 | 0.044 | 0.023 | 0.156 | 0.215 |

*Note.* UD, unipolar depression (patients with major depressive disorder whose diagnosis remained unchanged after follow-up); BD, bipolar disorder; tBD, patients who initially diagnosed with major depressive disorder transformed into BD during follow‐up; HAMD-17, 17-item Hamilton Depression Rating Scale; HAMA, Hamilton Anxiety Scale; YMRS, Young Mania Rating Scale; A, the left postcentral gyrus; B, the right inferior temporal gyrus; C, the left middle frontal gyrus; D, the right lingual gyrus; E, the right middle frontal gyrus; F, the left middle occipital gyrus; G, the right middle temporal gyrus; H, the left precentral gyrus; I, the left inferior parietal gyrus; J, the left inferior temporal gyrus. None of the results survived after FDR correction.
