# Supplementary material for: Identifying misdiagnosed bipolar disorder using support vector machine: feature selection based on fMRI of follow-up confirmed affective disorders
Source: Transl Psychiatry. 2024 Jan 8;14:9. doi: 10.1038/s41398-023-02703-z (PMC10774279; doi:10.1038/s41398-023-02703-z)
Supplement: Supplementary file 2 — Supplementary figure and table legends [file 41398_2023_2703_MOESM2_ESM.docx]

**Supplementary figure and table legends**

**Supplementary Figure 1. The ROC curve of SVM classifier performance in the public datasets.**

*Note.* ROC, receiver operating characteristics; AUC, area under the curve.

**Supplementary Figure 2. The mean ROC curve of SVM classifier performance in the training dataset.**

*Note.* ROC, receiver operating characteristics; AUC, area under the curve.

**Supplementary Table 1**

**The importance scores of the first ten regions by SHAP analysis.**

**Supplementary Table 2**

**Model performance of Variance, Pearson, ANOVA, and Chi-square feature selection methods.**

**Supplementary Table 3**

**Model performance of L1 regularization penalty term feature selection.**

**Supplementary Table 4**

**Model performance of Lasso classifier.**

*Note.* Lasso refers to Least Absolute Shrinkage and Selection Operator; AUC refers to area under the curve.

**Supplementary Table 5**

**Model performance of elastic net classifier.**

*Note.* AUC refers to area under the curve.

**Supplementary Table 6**

**The generalization performance of the classifier on external data.**

*Note.* AUC refers to area under the curve.

**Supplementary Table 7**

**Demographic and clinical characteristics of training and testing datasets.**

*Note.* UD, unipolar depression (patients with major depressive disorder whose diagnosis remained unchanged after follow-up); UD_training, UD patients assigned to the training dataset; UD_testing, UD patients assigned to in the testing dataset; BD, bipolar disorder; BD_training, BD patients enrolled in the training dataset; tBD_testing, patients who initially diagnosed major depressive disorder transformed into BD during follow-up and included in the testing dataset; HAMD-17, 17-item Hamilton Depression Rating Scale; HAMA, Hamilton Anxiety Scale; YMRS, Young Mania Rating Scale; FD, framewise displacement; **p* < 0.05 was considered a statistical difference; NA, not available.

a. Kruskal-Wallis H test

b. ANOVA test

c. Chi-square test

d. Fisher’s exact test

**Supplementary Table 8**

**Correlations between functional connectivity and symptom scales in the most contribution brain regions.**

*Note.* UD, unipolar depression (patients with major depressive disorder whose diagnosis remained unchanged after follow-up); BD, bipolar disorder; tBD, patients who initially diagnosed with major depressive disorder transformed into BD during follow-up; HAMD-17, 17-item Hamilton Depression Rating Scale; HAMA, Hamilton Anxiety Scale; YMRS, Young Mania Rating Scale; A, the left postcentral gyrus; B, the right inferior temporal gyrus; C, the left middle frontal gyrus; D, the right lingual gyrus; E, the right middle frontal gyrus; F, the left middle occipital gyrus; G, the right middle temporal gyrus; H, the left precentral gyrus; I, the left inferior parietal gyrus; J, the left inferior temporal gyrus. None of the results survived after FDR correction.
