# Supplementary figures and images for: Identifying misdiagnosed bipolar disorder using support vector machine: feature selection based on fMRI of follow-up confirmed affective disorders
Source: Transl Psychiatry. 2024 Jan 8;14:9. doi: 10.1038/s41398-023-02703-z (PMC10774279; doi:10.1038/s41398-023-02703-z)

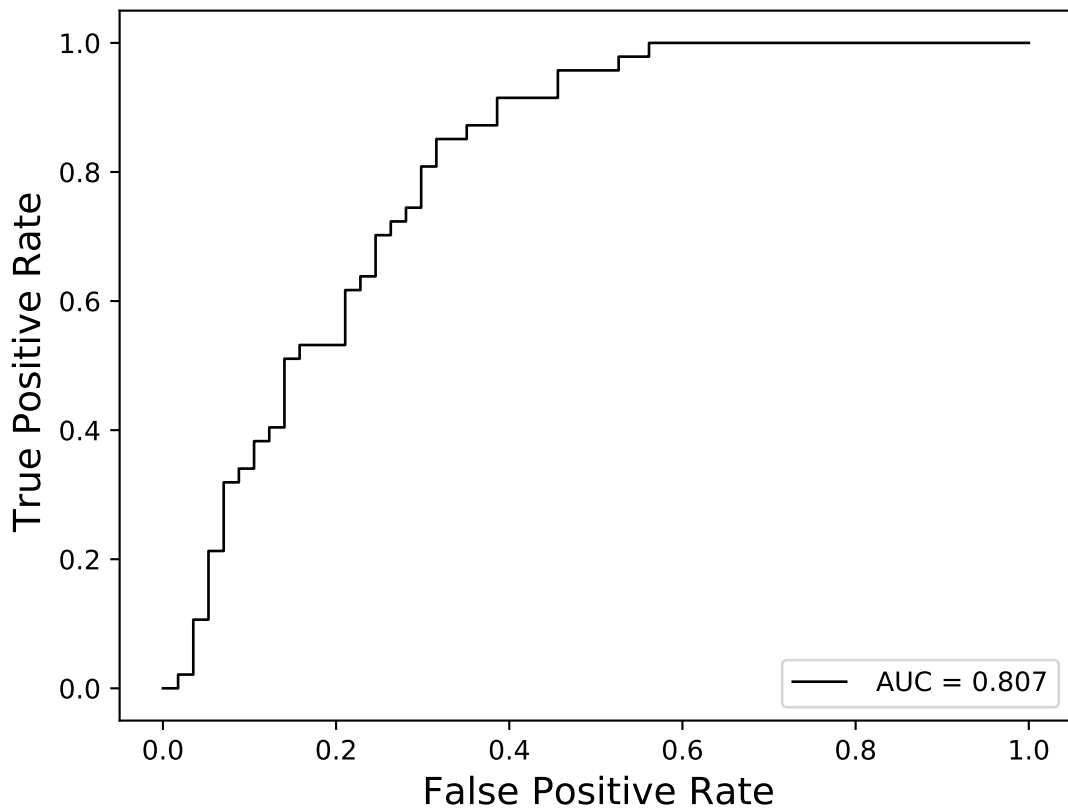

Supplement: Supplementary file 3 — Supplementary Figure 1 [file 41398_2023_2703_MOESM3_ESM.pdf]

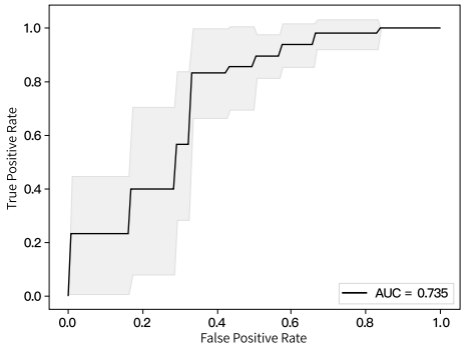

Supplement: Supplementary file 4 — Supplementary Figure 2 [file 41398_2023_2703_MOESM4_ESM.pdf]
